# A new mercury‐accumulating *Mucor hiemalis* strain EH8 from cold sulfidic spring water biofilms

**DOI:** 10.1002/mbo3.368

**Published:** 2016-05-13

**Authors:** Enamul Hoque, Johannes Fritscher

**Affiliations:** ^1^ Helmholtz Zentrum München – German Research Center for Environmental HealthInstitute of Groundwater EcologyIngolstädter Landstr.1Neuherberg85764Germany

**Keywords:** Aquatic fungus, Biofilm, mercury remediation, *Mucor hiemalis*EH8

## Abstract

Here, we report about a unique aquatic fungus *Mucor hiemalis* EH8 that can remove toxic ionic mercury from water by intracellular accumulation and reduction into elemental mercury (Hg^0^). EH8 was isolated from a microbial biofilm grown in sulfidic‐reducing spring water sourced at a Marching's site located downhill from hop cultivation areas with a history of mercury use. A thorough biodiversity survey and mercury‐removal function analyses were undertaken in an area of about 200 km^2^ in Bavaria (Germany) to find the key biofilm and microbe for mercury removal. After a systematic search using metal removal assays we identified Marching spring's biofilm out of 18 different sulfidic springs' biofilms as the only one that was capable of removing ionic Hg from water. EH8 was selected, due to its molecular biological identification as the key microorganism of this biofilm with the capability of mercury removal, and cultivated as a pure culture on solid and in liquid media to produce germinating sporangiospores. They removed 99% of mercury from water within 10–48 h after initial exposure to Hg(II). Scanning electron microscopy demonstrated occurrence of intracellular mercury in germinating sporangiospores exposed to mercury. Not only associated with intracellular components, but mercury was also found to be released and deposited as metallic‐shiny nanospheres. Electron‐dispersive x‐ray analysis of such a nanosphere confirmed presence of mercury by the HgM_*α*_ peak at 2.195 keV. Thus, a first aquatic eukaryotic microbe has been found that is able to grow even at low temperature under sulfur‐reducing conditions with promising performance in mercury removal to safeguard our environment from mercury pollution.

## Introduction

Due to worldwide emission, release, long‐distance transport, and biomagnification of highly toxic mercury, combating mercury is declared as a main goal of an important environmental program instigated by the United Nations' Organization (UN, 2013). The ubiquitous occurrence of mercury in the environment is mainly due to (1) extensive use of mercury‐containing fossil fuels (e.g., coal), (2) artisanal and small‐scale gold production, (3) direct use of mercury or mercury products in industry, agriculture, and medicine, (4) long‐distance transport of volatile zero‐valent mercury and methyl mercury, and (5) its biomagnification via the food web (Ribeyre et al. 1995; Pickhardt et al. 2005; UN, 2013). In 2010, the worldwide emissions of toxic mercury rose to about 1960 t increasing the background concentrations, interactions, and risk of mercury in the environment (UN, 2013). Therefore, understanding biological mercury transformation mechanisms and the development of appropriate mercury‐removal technology for contaminated water, sediments, soils, and air are considered to be important fields of research (Bryan and Langston 1992; Essa et al. 2002; Green‐Ruiz 2006; UN, 2013).

Mercury interacts not only with higher organisms but also with microbes. As compared to higher organisms, several microbe strains were shown to absorb, accumulate, and remove mercury, which led to worldwide awareness and research in the development of low‐cost bacterial mercury‐removal technology (von Canstein et al. 1999; King et al. 2002; Kiyono et al. 2003; Takeuchi et al. 2003). Although some expensive abiotic mercury‐removal technologies are commercially available, the low‐cost biological ones are still considered as important alternatives (Brim et al. 2000; Green‐Ruiz 2006). The existing biotechnologies mainly apply special bacteria (von Canstein et al. 1999; Green‐Ruiz 2006), genetically engineered bacteria (Kiyono et al. 2003; Zhao et al. 2005; Pfeiffer et al. 2012), biological precipitation technique (Pan‐Hou and Imura 1981; Aiking et al. 1985), biological reduction and volatilization (von Canstein et al. 1999; King et al. 2002; Takeuchi et al. 2003), biosorption by bacterial biomass (Saglam et al. 2002; Kiyono et al. 2003), and phytoremediation technology (Landigren et al. 2015). The accumulation of mercury as HgS in the cells of Rabbit‐foot grass (*Polypogon monospeliensis*) leaves with substantial biomass was suggested to be effective phytoremediation to cleanup mercury‐contaminated sites (Landigren et al. 2015).

In contrast, the absorption of mercury to microbial biomass does not necessarily require use of live cells (Saglam et al. 2002; Green‐Ruiz 2006) and its waste disposal is advantageous because of smaller amount needed. However, instead of mercury absorption, the intracellular mercury accumulation by live bacterial cells and the waste removal of internalized mercury could be far better and safer for handling, but this mercury‐removal mechanism is not widespread among bacteria. The bacteria *Klebsiella aerogenes* NCTC418 facilitated the precipitation of ionic Hg as HgS near the cell perimeter (Aiking et al. 1985). Lefebvre et al. (2007) detected accumulation of mercury as HgS in the cells of cyanobacteria. *Bacillus* sp. was shown to actively take up ionic mercury and subsequently reduce it into elemental metallic mercury using two enzymes; oragnomercury lyase and mercuric reductase (Schiering et al. 1991). Reduction of Hg(II) by some mercury‐sensitive dissimilatory metal‐reducing bacteria was also reported by Wiatrowski et al. (2006). Metallic mercury, if not volatilized, can be oxidized back into water‐soluble ionic mercury and thus easily distributed in the whole body of a living organism (Naidich et al. 1973) or transformed into methyl mercury by biological systems (UN, 2013) that exerts even stronger toxicity. Toxic ionic mercury and methyl mercury cations are known to interfere with sulfohydryl functional groups of many essential metabolic enzymes or cysteinyl proteins and to inhibit such enzymes or proteins in organisms (Naidich et al. 1973). Plasmids of gram‐positive and gram‐negative bacteria carry genes responsible for the expression of enzymes and proteins that confer cellular resistance and binding ability against organic and inorganic mercury (Hamlett et al. 1992). The *MerA* gene encodes the mercuric reductase, *MerB* the organomercury lyase for cleavage of mercury from organomercuric compounds, *MerR* the regulatory protein for mercury, *MerD* for downregulation, *MerP* for periplasmic binding and transfer, and *MerT* for cytoplasmic Hg(II)‐specific transport or directly for transfer to the mercuric reductase (Hamlett et al. 1992; Felske et al. 2003). Reduction of Hg(II) into elemental mercury by mercuric reductase encoded by the *MerA* gene confers mercury tolerance and resistance in bacteria. This is because of the fact that the resultant elemental mercury is either metabolically inactive or it can escape the microbe by volatilization (Hamlett et al. 1992). The mercury‐resistance determinants of plasmid PSB102, isolated from a microbial population residing in the rhizosphere of alfalfa, were found to be located on a transposable element of the TN5053 family‐designated *Tn5718* (Schneiker et al. 2001). Mercury applications enhanced transfer of the mercury‐resistance gene via self‐transmissible mercury‐resistance plasmids (Smit et al. 1998). Interestingly, a coselection of mercury‐resistance and antibiotic‐resistance genes in *Escherichia coli* takes place after application of mercury to bacteria (Skurnik et al. 2010).

Not only bacterial biomass, but also some terrestrial fungal biomass absorbs (Saglam et al. 2002) or accumulates (Kalac et al. 2004; Falandysz et al. 2015) mercury significantly. Several fungal strains were found to grow in mercury‐contaminated soil (Gajendiran and Abraham 2014), whereby they can accumulate mercury. Lodenius and Herraren (1981) reported about significant concentrations of mercury up to 200 mg/g dry‐wt. in higher fungi growing in the vicinity of chlor‐alkali‐plant in Finland, whereas in less‐polluted urban areas concentrations up to 64 mg/kg dry‐wt. were detected (Lodenius et al. 1981). Some edible fungi, mainly of the genera *Agaricus*,* Macrolepiota*,* Lepista, Calocybe* (Kalac et al. 2004), and *Boletus* (Falandysz et al. 2015), were found to accumulate mercury. Humus‐decomposing fungi accumulate generally more mercury than wood‐decomposing and some mycorrhizal ones (Bargagli and Baldi 1984). The growth of some ectomycorrhizal fungi (ECMF) was inhibited by mercury, whereby the biomass production by some ECMF was also lowered (Crane et al. 2010). About 95.3% of 100‐mg Hg(II) L^−1^ was adsorbed at pH 5.5 and 30°C by 1‐g *Mucor rouxii* IM‐80 biomass (Martinez‐Juarez et al. 2012). In the fungus *Aspergillus flavus* strain KRP1 the mercury(II) tolerance at a concentration of 100 mg L^−1^ was observed at pH 5.5–7 and 25–35°C (Kurniati et al. 2014). Volatile methyl mercury was mainly produced in the mycelium by *Aspergillus niger*,* Scopulariopsis brevicaulis*, and *Saccharomyces cerevisiae* (Vonk and Sijpesteijn 1973). The fungi *Aspergillus niger* and *Cladosporium* isolates volatilized almost 80% of initial mercury content during 7‐day static cultivation in the darkness (Urik et al. 2014). Fischer et al. (1995) demonstrated for the first time that two xenic cultures of saprophytic macromycetes *Coprinus comatus* and *Coprinus radians* were also able to methylate mercury.

In contrast to bacteria and terrestrial fungi with higher temperature optimum, we wanted to explore the possibility of there being any mercury‐removing aquatic fungi in biofilms of cold sulfidic spring water with special ability to grow even at lower temperature. Of six groundwater regions in Bavaria, Germany, three springs of the type sulfidic zinc‐hydrogen carbonate from the same karstic groundwater “Franconian Alb/Tertiary Hill” region were intensively investigated for mercury‐removal screening experiments by biofilms: (1) Sippenauer Moor with usual content of heavy metal ions and without any strong sign of anthropogenic influences because of its location in a nature conservation area, (2) Irnsing H_2_S with additional anthropogenic influence of nitrate and pesticides because of maize cultivation in the uphill regions (Hoque et al. 2007), and (3) Marching spring at present without any sign of anthropogenic influence, but located downhill of hop plant (*Humulus lupulus*) cultivation fields and thus can be influenced by the cultivation activities. Tritium measurements also exhibited mixing of additional young water from uphill regions apparently via surface runoff and porous rock infiltration into the spring water of Irnsing H_2_S and Marching. Agricultural records revealed that mercury was formerly used for the treatment of poles used for hop plant cultivation in the uphill regions of Marching spring that could have entered its water and created adaptation pressure to form a unique biofilm with a microorganism community resistant to mercury.

Therefore, we investigated water, biofilms, biodiversity, and aquatic fungi of the cold sulfidic spring Marching, especially concerning mercury concentration and its elimination. Thus, the objectives of our study were to (1) undertake a thorough systematic search for biodiversity, and mercury‐removal function analyses of 18 different cold sulfidic spring water biofilms, (2) to identify the key aquatic microbe/fungus for mercury removal from the efficient mercury‐removing biofilms, (3) to cultivate and assay the effectiveness of the key aquatic microbe/fungus in mercury removal, (4) to compare the biological mercury‐removal functions by kinetic analysis, and (5) to present a unique low‐cost and low‐waste biotechnology for rapid removal of mercury by aquatic fungus from mercury‐contaminated water even at low temperature.

## Experimental Procedures

### Site description, chemical, and physical parameters of sulfidic spring

The biofilm of mercury elimination was obtained from Marching spring, a cold sulfidic karst spring with a mean water temperature of 10.3°C and a sulfur content of 6.94 wt% located in Bavaria (Germany) close to the Danube river, 30 km southwest of Regensburg at geographical coordinates: latitude 48°49′12.78″ and longitude 11°42′55.65″. Chemical and physical parameters of Marching spring water were analyzed as previously described (Hoque et al. 2007). Mercury in spring water was additionally investigated by using high‐resolution quadruple ICP‐MS (inductively coupled plasma mass spectrometry) technique (Schramel and Wendler 1999). Water age was determined by using tritium and ^14^C‐analysis data as previously described (Heinrichs et al. 2000; Fritscher 2004).

### Biofilm collection and enrichment culture

Fresh thread‐like thin biofilms (2–5 g) floating near water surface were collected in sterile falcon tubes and immediately placed on ice. The biofilms were transported on ice to the laboratory and immediately centrifuged at 4000*g* (5 min) to give pellets. The pellets obtained were purified (Hoque et al. 2007) and either subjected to DNA extraction (see below) or used for other experiments (e.g., biofilm purification, fungus isolation, metal removal assays, metal content analysis; see below). Repeated reinoculations and cultivations of biofilm pellets on fungus‐selective malt extract‐agar solid medium (30:15 w/w; Arjmand and Sandermann 1985) led to the isolation of pure fungal cultures suitable for characterization and identification using various methods (see below). The ability of the mycelia of aquatic fungi from cold sulfidic springs to grow even at lower temperatures was tested as described previously (Hoque 2003).

### Biodiversity analysis and identification of fungi in biofilm

Biodiversity analyses of Marching spring water biofilms using fluorescence in situ hybridization (FISH) technique (Hoque et al. 2007), microscopic observations, and conventional isolation procedures were conducted. Subsequently, the fungal biodiversity and the identification of fungi in spring biofilms was carried out, employing a specific primer pair for amplification of the fungal ITS1‐5.8S‐ITS2 rDNA region, ITS cloning, and sequence analysis (see DNA extraction below). Identification of fungus based on habitat, morphology, crossing experiment, and fluorescence in situ hybridization (FISH) analysis was carried out as previously described (Hoque et al. 2007).

### DNA extraction from biofilm

DNA from EH8 and Marching biofilms (pellet ~500 *μ*L) was extracted according to the protocol described by Lueders et al. (2004). Extracted DNA was quantified, purity checked by using Nano‐Drop UV/VIS photometer (PEQLAB, Erlangen, Germany), and electrophoresis on 1% agarose gel and staining with ethidium bromide.

### PCR amplification of fungal‐specific genes

Fungal biodiversity analysis was carried out by fungal ITS1‐5.8S‐ITS2 amplification, cloning, and sequencing using extracted pure DNA. Two specific primer pairs capable of amplifying a ~600–650 bp fungal ITS rDNA region by PCR were tested for amplification efficiency: EF3RCNL (f) (5′‐CAA ACT TGG TCA TTT AGA GGA‐3′, reverse complement of EF3; Lord et al. 2002; Smit et al.^1999^) paired with ITS4 (r) (5′‐ TCC TCC GCT TAT TGA TAT GC‐3′; White et al. 1990), and ITS1F (f) (5′‐CTT GGT CAT TTA GAG GAA GTA A‐3′; Gardes and Bruns 1993) paired with ITS4 (r). The latter primer pair was selected and used on the basis of its higher PCR product yield. PCR amplification reactions (Lueders et al. 2004; modified) were carried out in 50 *μ*L with 1× PCR buffer, 1.5 mmol/L MgCl_2_, 10 *μ*g BSA, 0.1 mmol/L dNTPs, 0.5 *μ*mol/L forward primer, 0.5 *μ*mol/L reverse primer, 1 U Taq Polymerase (Invitrogen), and 1–20 ng template DNA. Thermocycling for PCR was run at 94°C for 3 min, 30 cycles of 94°C for 30 sec, 53°C (first primer pair) or 55°C (second primer pair) for 30 sec, 72°C for 1 min, followed by 72°C for 10 min, and final hold at 4°C. The PCR products obtained were purified on MinElute columns (Qiagen) and re‐eluted in 25 *μ*L eluting buffer (10 mmol/L Tris‐HCl, pH 8.5), quantified by using Nanodrop UV spectrophotometer (PEQLAB, Germany), and then analyzed by agarose gel electrophoresis and staining with ethidium bromide.

### ITS rDNA clone library construction

Optimal amounts of ITS amplicons obtained by using the ITS1F (f) and ITS4 (r) primer pair (see above) were cloned by T4 DNA ligation into pGEM‐T M13 vector and transformation of the vector plasmids into competent *E. coli* cells with pGEM‐T vector system II (Promega, Madison, USA). Single bacterial colonies containing plasmids with ITS rDNA inserts were identified by blue/white screening.

### Analysis of ITS rDNA clone libraries

Plasmid DNAs were isolated from randomly selected ITS clones and used as DNA templates for M13 PCR. M13 PCR reactions were performed in 50 *μ*L with 1× PCR buffer, 1.5 mmol/L MgCl_2_, 0.1 mmol/L dNTPs, 0.5 *μ*mol/L M13 forward primer, 0.5 *μ*mol/L M13 reverse primer, 1 U Taq Polymerase (Invitrogen), and 1–20 ng template plasmid DNA. Thermocycler parameters for PCR were set at: 94°C for 3 min, 23 cycles of 94°C for 30 sec, 55°C for 30 sec, 74°C for 90 sec, followed by 74°C for 5 min, and final hold at 4°C. The PCR products were analyzed by 1% agarose gel electrophoresis and staining with ethidium bromide, quantified by using Nanodrop UV spectrophotometer (PEQLAB, Germany), and then purified on MinElute columns (Qiagen) and re‐eluted in 25 *μ*L eluting buffer (10 mmol/L Tris‐HCl, pH 8.5).

### Cycle Sequencing

The cycle‐sequencing reactions for ABI Prism 3730 sequencer were performed in 5.5 *μ*L with reaction mixture (BigDye), 1.5 *μ*L sequencing buffer (stock solution 5×), 1 *μ*L terminator v3.1 premix, 1 *μ*L 10 *μ*mol/L sequencing primer T7f or M13 rev, and 2 *μ*L (8 ng) template (M13 amplicon, see above). The thermal protocol for cycle sequencing was set at: 96°C for 1 min, 25 cycles of 96°C for 10 sec, 50°C for 5 sec, 60°C for 4 min with slow temperature ramping (~1°C min^−1^), and final hold at 4°C. The cycle‐sequencing products were desalted by filtration on DyeEx spin columns (Qiagen) and sequenced by the gene core facility center (Helmholtz Zentrum München, Germany).

### Phylogenetic tree construction

ITS sequences were assembled using the DNASTAR laser gene software (v. 6, DNASTAR Inc., Madison, USA) and unambiguous ITS sequences were exported in FASTA format. ITS clone sequences were stored in an own ITS database along with other fungal ITS sequences downloaded from NCBI, then analyzed and aligned by Clustal W software (v.1.83, http://www.ebi.ac.uk/clustalw/). The alignments therefrom were exported in Nexus formats for phylogenetic tree construction by using Paup software (win‐paup4b10.exe, v. D. L. Swofford). The ITS sequences (see results) of the ascomycetes *Leucostoma personii* and *Aspergillus fumigatus* (Schüßler et al. 2001) and the zygomycetes *Basidiobolus ranarum* (Voigt et al. 1999) and *Scutellospora castanea* (Dodge and Wackett 2005) were selected as outgroup. Neighbor‐joining heuristic search method with parsimony optimality criterion followed by bootstrap analysis with 1000 replicates was employed for the calculation of phylogenetic tree in order to show the evolutionary relationships of aquatic *Mucor hiemalis* strain EH8 from sulfidic spring water to other known fungi. The phylogenetic tree was visualized by TreeView software (v.1.6.6, R. D. M. Page, Glasgow Univ., UK).

### Light microscopy and combined scanning electron microscopy–electron‐dispersive x‐ray (SEM‐EDX) analysis

The biofilms and the microorganisms therein as well as the pure fungal cultures obtained therefrom (see above) were observed by using stereo (model 16MZ, Leica) and phase contrast light (model Axiovert 100, Zeiss) microscopy after safranin staining (Hoque et al. 2007), as well as by using scanning electron microscopy (SEM; model JSM 630F, JEOL, Japan). For SEM's observations, the samples (biofilms, fungi) from PBS‐buffer (pH 7.4) suspensions were fixed at first with 1% glutaric aldehyde for 15 min and then with 2% osmium tetroxide in PBS buffer (pH 7.4). The fixed samples were dehydrated in an increasing gradient of ethanol (50%, 80%, and 100%). Then, the uncut samples were sprayed with gold nanoparticles prior to electron microscopic observations. The SEM was operated at 5–15 kV for secondary imaging, backscattered electron imaging, and electron‐dispersive x‐ray (EDX) analysis. The EDX system (Link eXL EDX system, Oxford Instruments, UK) for microanalysis was run in combination with SEM, and comprised an x‐ray detector, that is, an EDX detector in order to detect and convert x‐rays into electronic signals, a pulse processor in order to measure the electronic signals for the determination of the energy of each x‐ray detected, and an analyzer in order to display and interpret the x‐ray data. The main components of the x‐ray detector used were a collimator assembly to limit the x‐ray aperture, an electronic trap (permanent magnet filter) to deflect highly energetic x‐rays, a window barrier for vacuum maintenance, a Si(Li) semiconductor crystal to detect x‐rays and to produce proportional electric charge signals, and a FET (field effect transistor) to measure and preamplify the electric charge signals produced by the detector crystal. The preamplified voltage signals produced by FET of the EDX system during microanalysis of biofilms and EH8 spores were fed to the pulse processor to count the x‐ray energy pulses, then interpreted and displayed as counts per second versus energy pulse level (keV, energy channel) by the analyzer using Link Analytical Software (Oxford Instruments, High Wycombe, UK).

### Mercury analysis

#### Mercury stock solutions

Heavy metal salts, for example, HgCl_2_, of analytical grade were obtained from commercial sources (Sigma‐Aldrich, Taufkirchen, Germany; Merck, Darmstadt, Germany). Stock solutions of the required concentrations were prepared in ultra‐pure de‐ionized water within the solubility limit of these metal salts.

#### Activation of EH8 sporangiospores

Sporangiospores from five EH8 (ca. 3 weeks old, grown on malt‐extract‐agar) culture plates were collected in 45‐mL sterile PBS (20 mmol/L phosphate‐buffer, 150 mmol/L NaCl, pH 7.4), purified and concentrated by repeated centrifugations (4000*g*, 10 min), and washed in PBS (pH 7.4) to pellets (Hoque et al. 2007). The spores were counted using a hemacytometer (Sigma) and image processing software (Proimage v.3.01, MicroMotion, Mainz, Germany). The physiological activation of 5 × 10^7^ (~28 mg dry‐wt.) sporangiospores was induced by incubation in 50‐mL C‐, N‐enriched medium (Kirk et al. 1978) at 30°C under shaking of 120 rpm. Activated spores were purified by repeated washing with PBS (pH 7.4) and centrifugation cycles, and the biomass yield (fresh and dry‐wt.) from aliquots was determined. The absorptions (A_270 nm_ and A_650 nm_) of diluted spore suspensions before and after activation were also determined. The calibration curves for spore count or from that spore biomass versus absorbance were calculated by regression analysis, and used to determine required amount (weight) of activated spore biomass for treatment of a particular water volume, as for example, by using the equation: *y* (mg, spore biomass dry‐wt.) = 12.26 + 8.096 × A_270 nm_ (*r* ^2^ = 1; 95% confidence intervals).

#### Mercury‐removal assay

As only biologically live, medium‐free, purified materials of Marching spring and EH8 therefrom showed significant mercury‐removal ability in contrast to dead sterilized materials, the mercury‐removal assays were intensively performed with live purified materials. In order to avoid effects of medium, cellular components, and biosorptions (Chang et al. 1993; Hintelmann et al. 1993), the metal‐removal assays using purified biofilms and EH8 were conducted in pure Munich groundwater only for short durations (max. 48 h). For comparison of mercury elimination performances, purified live medium‐free germinated spores of the terrestrial strain (DSM 2655) of the same fungus were also tested. Pure viable germinated spores (5 × 10^7^) of EH8 were suspended in at least triplicate in 50‐mL Munich groundwater (pH ~7) at 25°C containing different concentrations (100 *μ*g L^−1^, 1000 *μ*g L^−1^, 10,000 *μ*g L^−1^, and 50,000 *μ*g L^−1^) of Hg(II) and shaken at 120 rpm for 48 h in closed falcon tubes. After 48 h, the whole suspensions were centrifuged (4000*g*, 5 min, 4°C; Megacentrifuge 1.0R, Heraeus) to separate supernatant and pellets (spore biomass). The aliquots of supernatants were acidified with hydrochloric acid, and diluted with demineralized water to 10 mL. Specimens of live biomass (pellet) were observed after preparation by using SEM. Remaining biomass was dissolved by covering it with concentrated HNO_3_ and diluted to 10 mL before measurement using an inductively coupled plasma detector (ICP, Model Liberty 200A, Varian). In order to increase ICP measurement sensitivity, a hydride generator (VGA 76, Varian) was used, whereby the aliquots of dissolved samples were treated initially with HCl and then reduced with sodium hydroxide‐stabilized NaBH_4_. A stream of Argon was used to direct this liquid mixture into a reaction coil to generate gaseous mercury hydride. The volatile hydrides formed were separated from liquid phase at a liquid/gas separator, transferred by a second argon stream into the plasma of the ICP detector, and the intensities of the mercury‐specific line at 194.163 nm were measured. The system was calibrated by using standards of 0, 10, 100, 500, and 1000 *μ*g L^−1^ Hg(II) in acidified Munich groundwater, whereby the calibration curves were calculated by linear regression analysis (*r* ^2^ = 0.99) with 95% confidence intervals.

#### Analysis of mercury in spring water and biofilm by ICP‐MS

In order to determine very low concentrations of mercury in spring water and biofilms, the highly sensitive inductively coupled plasma–mass spectrometry (ICP‐MS) method of Schramel and Wendler (1999) with a typical detection limit of 0.15 pg was used. The supernatants and biofilm biomass (pellet) were separated by centrifugation. The supernatants (spring water) acidified with 250 *μ*L conc. HCl and the pellets digested with conc. nitric acid were diluted to 10 mL prior to analysis of aliquots by ICP‐MS.

#### Effects of pH on mercury‐removal efficiency

The effects of pH on mercury removal (%) by activated EH8 sporangiospores (5 × 10^7^ cells) in groundwater were investigated at pH 4, 6, 8, and 9 after an exposure of 48 h to 1000 *μ*g L^−1^ Hg(II). Standard deviations (*n* = 3) across all data points were not more than  ± 5%.

#### Removal of mercury by EH8 in bulk water

Increasing volumes (10–100 L) of groundwater containing 1000 *μ*g L^−1^ Hg(II) were treated with increasing biomass (0–97.9 g, dry‐weight) of germinating EH8 spores in a nylon net. After 48‐h incubations, the concentrations of mercury in water phase and spore biomass following centrifugation were determined. The calibration curves were calculated to determine the required amount of biomass (g, dry‐weight) depending on volume of contaminated water by linear regression analysis (*r* ^2^ = 1) with 95% confidence intervals.

### Statistical analysis

The statistical analysis of data, especially curve fitting by regression analysis, was performed by using Sigma Plot software for windows (v. 8.02, SPSS, Chicago, USA). Student's t‐tests and nonparametric Mann–Whitney U‐tests were performed according to Weber (1986).

### Strain deposit

The pure culture of aquatic *M. hiemalis* strain EH8 was deposited at the DSMZ (Germany) under the accession number DSM 16290.

### Nucleotide sequence accession numbers

Sequences (18S rRNA gene, partial sequence; internal transcribed spacer 1, 5.8S rRNA gene, and internal transcribed spacer 2, complete sequence; and 28S rRNA gene, partial sequence) of *M. hiemalis* f. *hiemalis* strain EH8 (plus‐strand) and *M. hiemalis* f. *hiemalis* strain EH8r (minus strand) from Marching biofilm DNA identified in this study were deposited in GenBank under accession numbers GU183689 and GU183690, respectively.

## Results and Discussion

### Hydrogeology, hydrogeochemistry, and ecology of Marching spring

**Figure 1:**
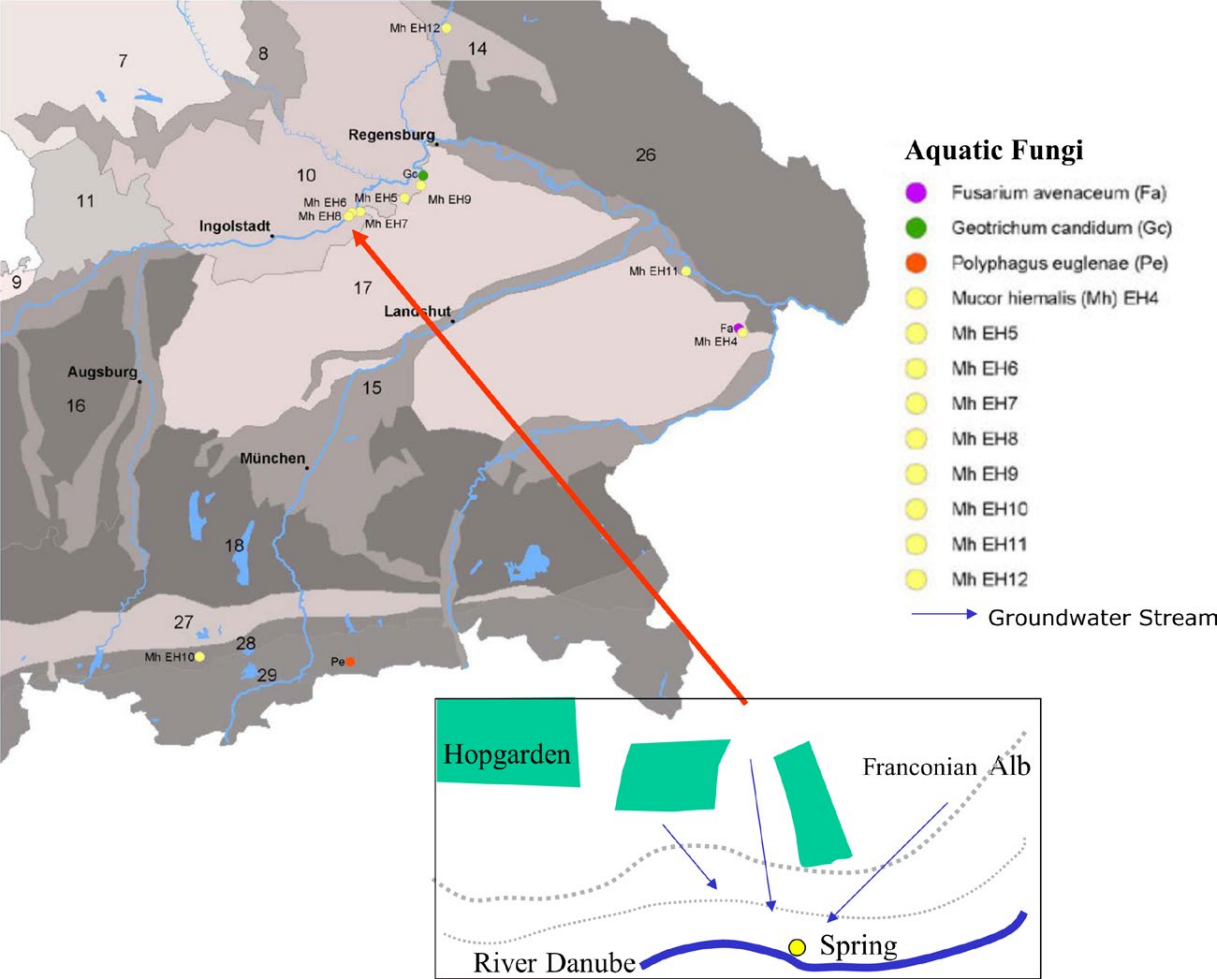
Topographic location of Marching spring among other sulfidic springs containing aquatic fungi in Bavaria. The spring with EH8 (see red arrow) is located downhill from the hop cultivation land (see exploded view, green areas below) with past mercury‐use history, specifically in the Great Hallertau Hopgarden areas.

**Figure 2:**
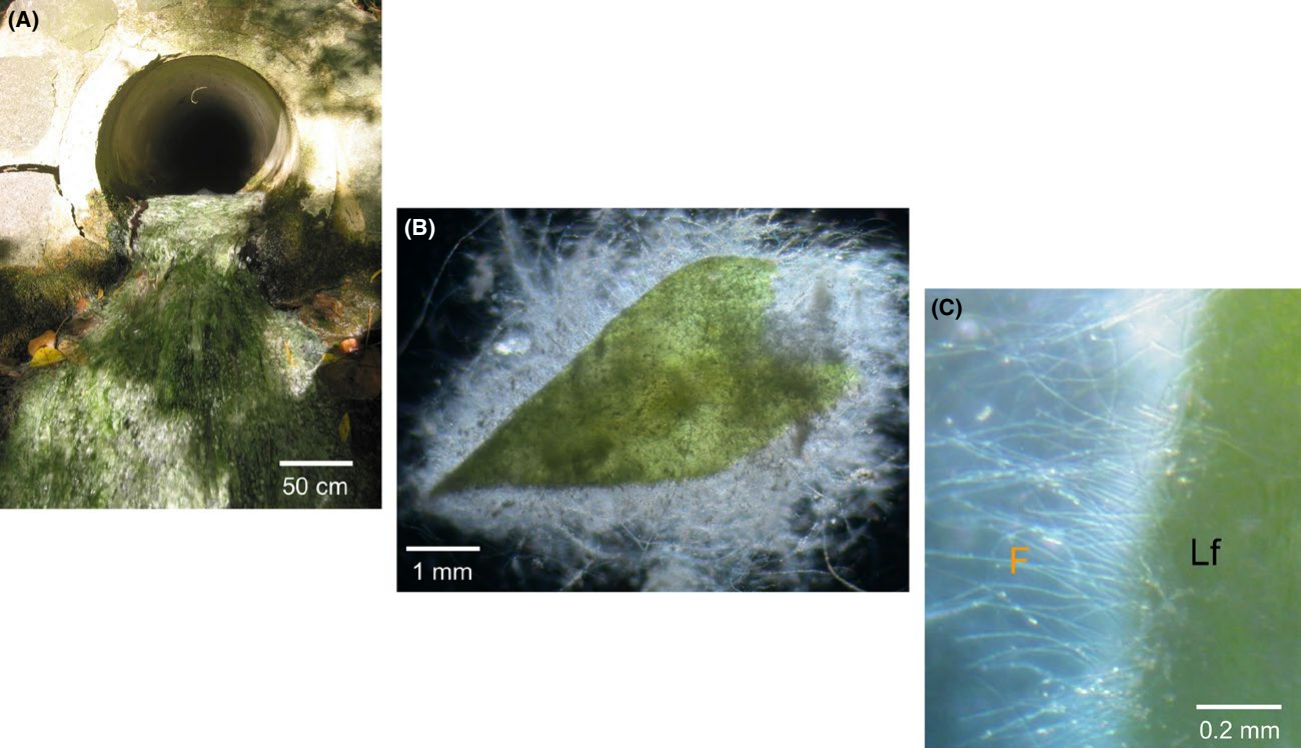
Association of biofilm with moss in the spring water of Marching. (A) Spring Marching with moss–microbial biofilm, (B) Moss *Brachythecium rivulare* (green part) is interfaced with the microbial biofilm (white part), and (C) microbial biofilm with fungus‐like filaments (F) interfacing moss leaf (Lf) after magnification using stereo microscopy.

The spring Marching is located at the southern, steep side of a valley at 330 m above sea level, downhill of former *Humulus lupulus* cultivation areas where mercury‐treated poles were used (Bavaria, Germany; Fig. 1), and belongs to the irregular limestone‐flowing spring (karstic rheokrene) type (Heinrichs et al. 2000). This irregular limestone is porous and can therefore store substances infiltrating from uphill for a long time, months to years, and then release them slowly during water discharge through the spring downhill. Marching spring's water falls at an angle of 30 degree from the southern steep side, flows through a pipe beneath a street (Fig. 2A) at a rate of about 120 L min^−1^, and after 30 m reaches a tributary of the Danube River. At the effluent end of the pipe, the thin thread‐like biofilm with microbial consortium floats in sulfidic‐reducing spring water (Eh ≤−173 mV, Table 1). During summer vegetation time it is exposed to intensive solar radiation and forms a biofilm–moss carpet (Fig. 2 B and C), initially 0.20 m broad × 4 m long, finally 1.20 m broad × 4 m long at the end of vegetation period. The moss was identified as *Brachythecium rivulare*. The biofilm–moss interface (see above) was continuously flushed with fresh sulfidic‐reducing spring water. Use of tritium analysis data in a dispersion model dated the younger part of water age to about 140 years (Heinrichs et al. 2000), a mixture of younger karstic water from the northern Franconian Alb, and older deep groundwater flowing through geological disturbance lines from a deep water molassic bed aquifer in the south. Marching spring's water contained some organic (e.g. nitrate) and inorganic (metal ions) pollutants beside sulfide (Table 1). The characteristic physical and chemical parameters of Marching spring's water as determined by ICP technique are given in Table 1. However, using sensitive ICP‐MS technique additionally, Al (4.52 ± 1.82 *μ*g L^−1^), Cr (<5.52 ± 0.03 *μ*g L^−1^), Ni (5.47 ± 0.04 *μ*g L^−1^), and Hg (14.3 ± 7.02 ng L^−1^), as well as P (<9.41 *μ*g L^−1^) were also detected in the water.

**Table 1:** Chemical and physical data of cold sulfidic Marching spring water. The chemical and physical data of the Marching spring are given below (September 2002). Some important contrasting chemical data are shown in bold face

Parameters	Springs	Marching
Spring discharge^a^		120 L min^−1^
Temperature		10.2–10.6°C
Electrical conductivity		623–672 *μ*S cm^−1^
pH		5.9–6.5
Redox potential (E_h_)		−173 to–185 mV
Oxygen		1.3–1.8 mg L^−1^
H_2_S		<1 mg L^−1^
Cations and total metals		
Na^+^		6.0–8.2 mg L^−1^
K^+^		0.7–1.1 mg L^−1^
Mg^2+^		31.3–31.5 mg L^−1^
Ca^2+^		81.2–85.9 mg L^−1^
Mn^2+^		4.5–4.9 mg L^−1^
Ba^2+^		15.5–20.4 *μ*g L^−1^
Total Co		<1 *μ*g L^−1^
**Total Cu**		**3.2**–**6.0 ** ***μ*** **g** L^**−1**^
Total Fe		1.9–7.8 *μ*g L^−1^
Li^+^		6.2–7.9 *μ*g L^−1^
Sr^2+^		78.9–117.2 *μ*g L^−1^
**Zn** ^**2+**^		**367.1**–**437.6 ** ***μ*** **g** L^−1^
Anions:		
**Total S** ^**2−**^		**0.4–0.6 mg L^−1^**
Cl^−^		11.5–12.1 mg L^−1^
** NO**_**3**_^**−**^		**0.1–1.1 mg L^−1^**
SO_4_^2−^		36.6**–**37.5 mg L^−1^
HCO_3_^−^		360.0**–**360.5 mg L^−1^
DOC^b^		1.1–1.5 mg L^−1^

a Varies depending on metereological parameters.

b Dissolved Organic Carbon.

### Enrichment of metal ions in Marching spring

Simultaneous sampling and measurement of metal ions in separated water phase and biofilm pellets of cold sulfidic spring water–biofilm suspensions showed a high enrichment of metal ions in live biofilm phase compared to the water phase (Hoque et al. 2007). Specifically, mercury was enriched in Marching spring water–biofilm (~23.91 ± 8.78 *μ*g/kg wet‐weight) as detected by ICP‐MS. Calculations showed high metal‐enrichment factors (= g metal/kg biofilms divided by g metal L^−1^ spring water) in Marching spring water–biofilm relative to concentrations of metal ions in spring water, for example, Al 1.6 × 10^4^, Cd 5.3 × 10, Cr 1.7 × 10^2^, Cu1.8 × 10^2^, Fe 5.8 × 10^4^, Li 2.6 × 10, Hg 3.26 × 10^3^, Mn 1.2 × 10^4^, Sr 8.2 ×,  and Zn 3.9 × 10, even though they were present at trace concentrations in inflowing spring water. In contrast to the fungus‐containing biofilms of 11 other sulfidic springs (Fig.1), the biofilm of the sulfidic Marching spring grew at the edges of moss (*Brachythecium rivulare*) leaves and showed microscopically fungus‐like filamentous‐mycelium structures in the biofilm (Fig. 2).

Although *B. rivulare* is known as an efficient heavy metal (Cd, Cr, Cu, Fe, Mn, Ni, Pb, and Zn) accumulator (Sanchez et al. 1998), the rapid mercury‐removal function of Marching spring's moss–biofilm interface was found to be confined only to the fungus‐like biofilm portions, not to the moss.

### Biodiversity: a thorough screening

Eighteen springs of Bavaria were thoroughly investigated concerning chemical and physical composition of water, mercury‐removal function, and biofilm types, whereby four biofilm types were differentiated. In the biofilms of nine springs we found different aquatic strains of *M. hiemalis* (EH 5, Hoque et al. 2007; EH 4, EH6–EH12) and in biofilms of three other springs we detected three different fungi (Fig. 1), and screened them all for mercury‐removal efficiency.

**Figure 3:**
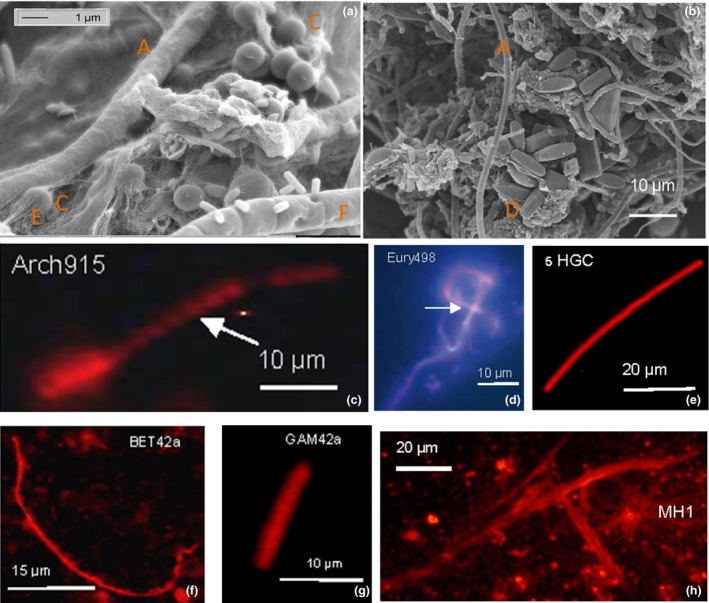
Biodiversity of Marching spring biofilm shown by electron microscopy and fluorescence in situ hybridization (FISH). (a, b) Electron microscope images of Marching biofilm showing (a) occurrence of, for example, coccoid archaea/bacteria (C), exopolymeric structures (EPS) structures (E), fungal hypha EH8 (F), and (b) diatoms (D), microalgae (A); (c–e) FISH labeling of (c) coccoidae archaea (Arch915), (d) euryarchaeota (Eury498), (e) high G‐C content bacteria (HGC), (f) ß‐proteobacteria (beta42a), (g) *γ*‐proteobacteria (GAM42a), and (h) *Mucor hiemalis* EH8 (MH1) with spores are shown. The FISH‐probes applied are denoted in c–h and in legend (see above and Experimental Procedures).

After finding that the Marching biofilm had the unique property of mercury removal, we thoroughly investigated its biodiversity from macro‐ to nanoscale. In the macroscale we found macrozoobenthos (*Asellus aquaticus*,* Elmis maugetii*, and *Lumbricus* sp.), in the microscale some aquatic fungi, microalgae, archaea, bacteria, and diatoms, and in the nanoscale we detected EPS (exopolymeric structures) and glutathione‐S‐transferase‐associated proteins (Hoque et al. 2003, 2007). Twenty one diatom species were found in the Marching spring biofilm, for which a saprophic value of 2 and a trophic value of 2.2 were determined. The biodiversity of Marching spring biofilm was analyzed by using 3‐D microscopy, phase contrast, and electron microscopy, as well as by FISH and other molecular biological techniques. The composition of Marching spring biofilm as revealed by SEM and FISH data images is exhibited in Figure 3.

SEM showed presence of coccoidae archaea and *M. hiemalis* (Fig. 3a), which were also confirmed by FISH analysis. Numerous diatoms and microalgae in the Marching biofilm were observed by SEM (Fig. 3b). FISH analysis revealed the composition of the Marching biofilm consortium as about 15% euryarchaeota, 5% *α*‐proteobacteria, 15% ß‐proteobacteria, 10% *δ*‐proteobacteria, 20% *γ*‐proteobacteria, 10% cytophaga and flavobacteria, 5% high G‐ and C‐rich bacteria, and 20% fungus *M*. *hiemalis* (see below, Fig. 3c–h). Later, the fungus *M. hiemalis* was isolated from this biofilm–moss leaf interface (Fig. 2), purified as a single pure culture, and identified as *M. hiemalis* f. *hiemalis* strain EH8 (plus strand). EH8's identification was at first based on (1) comparative morphology with plus strand (DSM 2655), (2) mating experiment with corresponding minus strand (DSM 2656; DSMZ Germany), as described for EH5 (Hoque et al. 2007), and (3) challenges with other aquatic *M. hiemalis* strains. Some other physiological and morphological reactions of challenges (4), for example, by fungal antagonism/toxicity tests (Holdenrieder 1982; Hoque 2003), differentiated EH8 from many other *M. hiemalis* strains. Later, molecular biology techniques were further used for its identification and phylogenetic tree construction, cloning and sequencing of ITS1‐5.8S‐ITS2.

rRNA gene using Marching biofilm DNA led to a 687 bp long sequence for comparison by bioinformatics tools. A search of the NCBI nucleotide database using BLASTN software (Altschul et al. 1997) showed a 99% similarity of 627 bp (major sequence portion) of 687 bp from Marching biofilm ITS clones (spring clone alias EH8) with the corresponding 628 bp region of *M. hiemalis* f. *hiemalis* CBS 242.35 supporting its identity as *M. hiemalis* f. *hiemalis* (plus strand).

**Figure 4:**
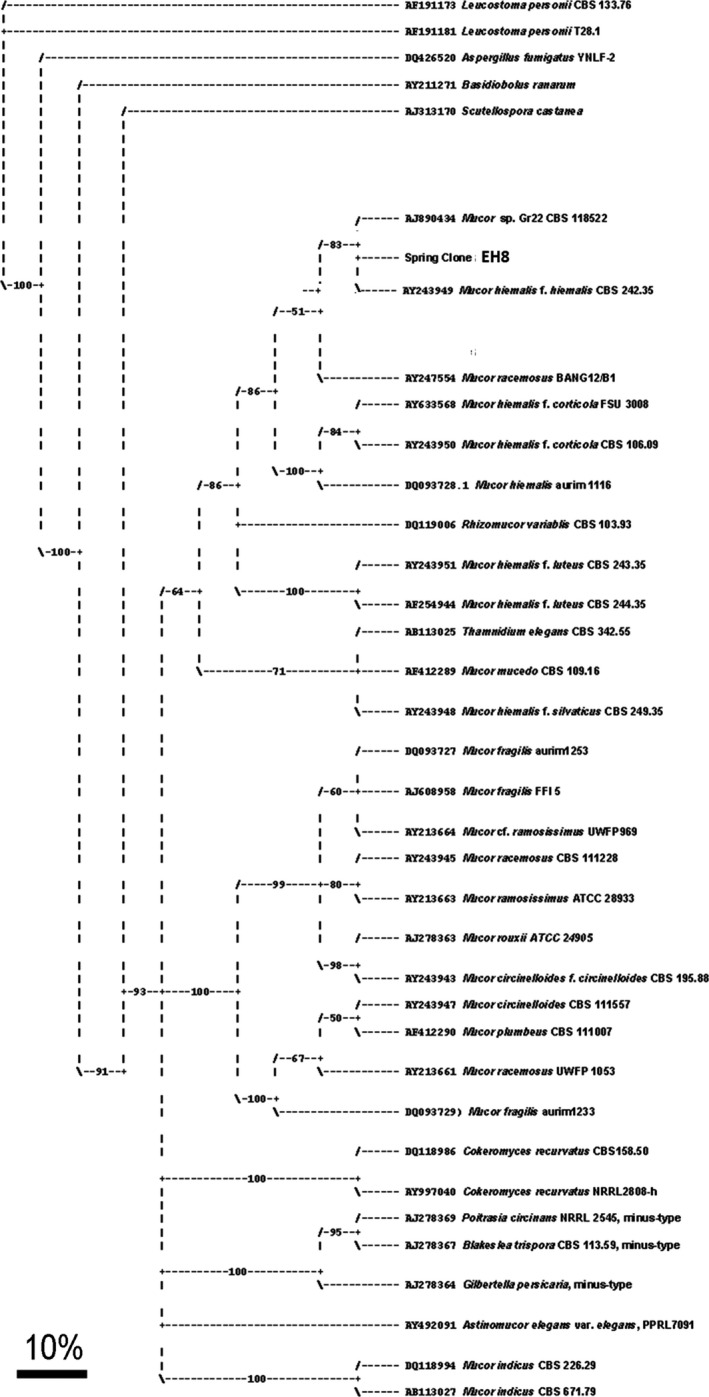
Phylogenetic tree based on ITS1‐5.8S‐ITS2 sequence data showing the position of aquatic *M. hiemalis* EH8 (Spring clone EH8) from the sulfidic‐sulfurous Marching spring biofilms in comparison to related fungal strains and some other known fungal species of the class zygomycetes (see Experimental Procedures). Bootstrap values greater than 50% are shown at the nodes. Bar = 10% estimated difference in nucleotide sequences.

The phylogenetic tree construction also revealed identity of spring clones (EH8) with *M. hiemalis* f. *hiemalis* plus strand (Fig. 4). However, the remaining nonaligned 60 bp of ITS1‐5.8S‐ITS2 rRNA gene sequence in EH8 might be different from other *M. hiemalis* f. *hiemalis* strains. Evidence of in situ occurrence of EH8 as *M. hiemalis* strain was provided by phase contrast microscopy, similar to EH5 (Hoque et al. 2007), as well as by SEM of microbial consortia (Fig. 3a).

### Mercury‐removal biofilms and identification of key mercury‐removal microorganism

Mercury‐removal screening assays led to the identification of Marching spring biofilm as the only biofilm consortium capable of mercury removal. The biofilms of the 17 remaining sulfidic springs did not contain the unique strain of EH8 and failed to remove mercury from water within 48 h. Earlier studies showed that the dead material from Marching spring biofilm and EH8 could not remove ionic mercury by biosorption. Thus, the live EH8 in the fungus‐like Marching biofilm could be suggested to be a crucial factor in removing mercury from water. Fungus‐like filament networks in biofilms as shown by 3‐D stereo microscopy appeared to provide physical support to the biofilm consortium.

### In situ function and the mercury‐removal capacity of EH8

The removal efficiency of biofilms and EH8 from Marching spring for mercury and zinc closely matched each other (see text below). The significant abundance (20%) of EH8 in Marching spring's microbial consortium could be an important factor leading to the major in situ group‐IIb heavy metal (Hg, Zn)–removal ability of biofilms of the same spring, as for example, the Hg‐ and Zn‐removal values of purified Marching biofilms (Hg 97.5%, see below; Zn 48.1%, Tab. S1) and its fungus EH8 (Hg 99.8%, see below; Zn 48.2%, Tab. S1) resembled each other. However, there was no similarity found for the removal efficiency of another group‐IIb metal Cd (Biofilm: 97.5%, EH8: 6%). In contrast, a preferred removal of mercury was shown to be associated with the active uptake and binding of the group‐IIb metal Cd to a metal‐binding motif coded by the same gene in *E. coli* (Pazirandeh et al. 1998). In this case, *E. coli* showed similarly strong accumulation of both Hg and Cd for metal concentrations of up to at least 2 *μ*mol/L. It holds true also for *Klebsiella aerogenes* NCTC 418 with Hg and Cd accumulations and depositions as metal sulfide and/or phosphate at the cell perimeter (Aiking et al. 1982, 1985). Thus, the physiological mercury‐removal mechanisms of EH8 and known bacteria can be different, but can also be mediated by similar metal reductase genes as found in both bacteria (Schiering et al. 1991) and fungi (Tezuka and Someya 1990; Baojun et al. 1995). Aquatic *M. hiemalis* strains from cold sulfidic springs were able to grow even at temperatures as low as 0.3°C (Hoque 2003).

**Figure 5:**
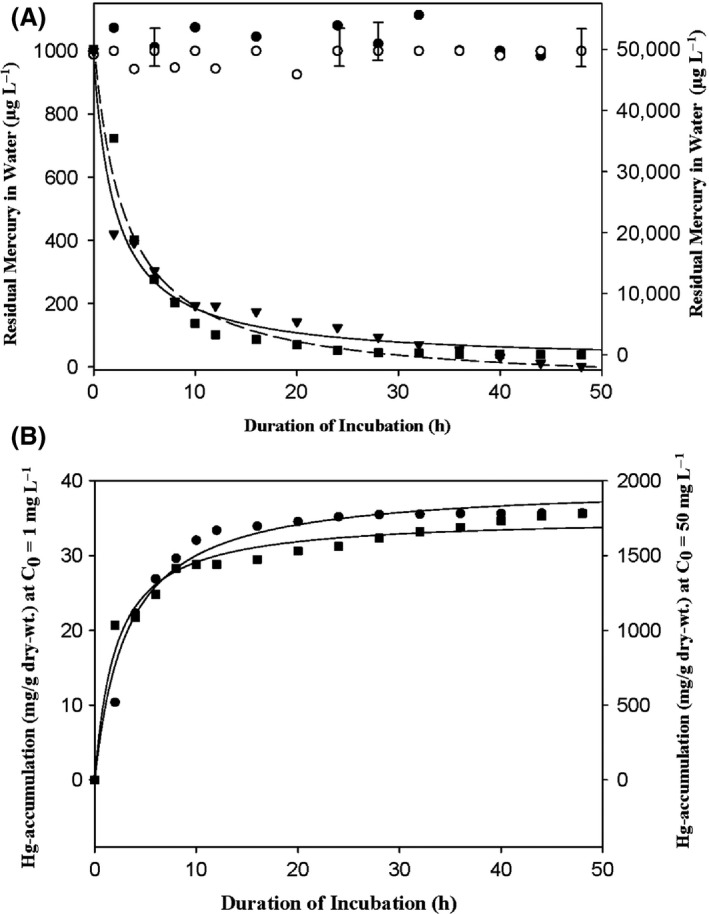
Decrease in ionic Hg concentration in water with concomitant intracellular Hg accumulation in *M*. *hiemalis* EH8 as a function of incubation time (h). (A) Decrease in Hg(II) concentration in water by *M. hiemalis* f. *hiemalis* EH8 (DSM 16290) at initial concentration (C_0_) of 1000 *μ*g L^−1^ (solid triangle) and 50,000 *μ*g L^−1^ (solid square), respectively, as compared to terrestrial strain *M. hiemalis* f. *hiemalis* DSM 2655 (open circle) and control values without fungus (solid circle). (B) Hg accumulation (mg/g dry‐wt.) by activated (germinated) spore biomass for two initial (C_0_) concentrations, 1 mg L^−1^ (solid square) and 50 mg L^−1^ (solid circle). Standard deviations at each data point were maximal ± 5%.

In contrast to aquatic EH8, other aquatic live *M. hiemalis* strains from eight other sulfidic spring water biofilms (Fig. 1) and the phylogenetically related terrestrial *M. hiemalis* f. *hiemalis* strain DSM 2655 failed to remove mercury from water as the residual mercury remained as high as control values (Fig.5A). These results suggest EH8 to be a new aquatic, eukaryotic, and low‐temperature adapted strain consecrated with unique mercury‐removal ability.

### Kinetics and mechanism of mercury removal by EH8 and mass balance

Numerous sporangiospores from EH8 sporangia released on solid malt‐extractagar medium were collected, purified, and activated during germination for high protein expressions (see Materials and Methods; Hoque et al. 2007). Intact activated medium‐free sporangiospores were incubated in nutrient‐free groundwater to avoid any unspecific mercury removal through interactions with a nutrient‐enriched medium (Chang et al. 1993; Hintelmann et al. 1993). Additionally, the time span was kept very short (48 h) in our mercury‐removal assays in order to minimize unspecific mercury removal due to cellular absorptions that might occur during longer incubation times (e.g., 140 h, Chang et al. 1993; 14;  days, Hintelmann et al. 1993). The kinetics of mercury removal by activated medium‐free EH8 spores in groundwater showed a rapid removal in the first 10 h reaching ca. 80% of the applied 50,000 *μ*g L^−1^ Hg(II) at an average clearance rate of ca. 67 *μ*g min^−1^(Fig. 5A). Within the next 11–48 h the removal rate decreased to ca. 4 *μ*g min^−1^. Note that intracellular Hg accumulation in both curves shows saturation‐like effects within the initial 10 h of incubation. Not only dead but also the activated medium‐free live spores of (1) the terrestrial strain DSM 2655 of the same fungus *M. hiemalis* f. *hiemalis* (Fig. 5A), (2) the other aquatic *M. hiemalis* strains, and (3) fungi isolated from other sulfidic springs failed to remove any significant amount of mercury from water. Even after only 2 h incubation time the mercury removal by aquatic EH8 was statistically significantly higher than that of the terrestrial *M*. *hiemalis* strain DSM 2655 (*P* ≤ 0.005, Student's one‐sided t‐test). Surprisingly, the EH8's effective mercury‐removal capacity of ~ 50,000 *μ*g Hg L^−1^ (Fig. 5A) and 1,977.018 mg Hg/g dry‐weight (recalculated B_max_, see below) exceeded that (7000 *μ*g L^−1^) of the bacteria‐based biotechnology (von Canstein et al. 1999) and the maximum mercury biosorption (287.43 mg Hg/g dry‐weight mycelium) by immobilized fungus *Pleurotus sapidus* (Yalcinkaya et al. 2002), respectively. Even during germination EH8 was found to be highly resistant to mercury, at least up to 50,000 *μ*g L^−1^, probably due to a mechanism described below. The mercury‐resistance phenomena were observed in two terrestrial fungi *Penicillium* sp. MR‐2 strain (Tezuka and Someya 1990) and *Cephalosporium tabacum* strain F2 (Baojun et al. 1995) as well as in bacteria *Acidithiobacillus ferro‐oxidans* SUG 2‐2 cells (Takeuchi et al. 2003) and *Pseudomonas putida* strains (von Canstein et al. 1999). In contrast, nonviable *Bacillus* sp. removed only 6.8–9.2% (recalculated) of initial mercury concentrations (0.25–10 mg L^−1^) that could be due only to biosorptions (Green‐Ruiz 2006). At pHs lower or higher than the physiological optimum pH ~7, the mercury fixed by EH8 showed only negligible release (see results). The mass balance analysis showed >99% mass recovery of applied Hg(II) up to 50,000 *μ*g L^−1^.

The EH8‐mediated decrease (y, *μ*g L^−1^) of initial mercury concentrations (C_0_) 1000 *μ*g L^−1^ and 50,000 *μ*g L^−1^ can be described by the three‐parameter rational functions; *y* = (983.2061 + 6.4927 *x*)/(1 + 0.4656 *x*) (*r* ^2^ = 0.97) and *y* = (51702.8459 − 1750.3206 x)/(1 + 0.3329 *x*) (*r* ^2^ = 0.98), respectively (Fig. 5A), whereby time‐dependent saturation‐like effects of accumulation took place after 10 h of uptake (Fig. 5B). It can be generalized that the kinetics of removal of mercury by EH8, that is, rapid decrease in mercury in water in the initial 0–10 h (Fig. 5B) and the slow decrease in the subsequent >10–48 h (Fig. 5), follows a best‐fit three‐parameter (a, b, and c) rational function of the type

(1) $$y\;=\;\frac{\left(a\;+\;b\;\times \;x\right)}{\left(1\;+\;c\;\times \;x\right)}$$

where *y* = concentration of metal (*μ*g L^−1^) in water phase, *x* = duration (h) of incubation, and 1 +  c × *x* ≠ 0 (Fig. 5A). Student's t‐test showed the two parameters (*a* and *c*) to be highly significant (*P* ≤ 0.0001), whereas the parameter b was significant (*P* ≤ 0.07) in most cases.

Active intracellular accumulation of mercury can be mediated by mercury transporter protein (*merT*) known to occur in bacteria (Hamlett et al. 1992), but it is not known in fungi (Thilakaraj et al. 2006). However, some P‐type ATPases may also be involved in fungi to pump ionic heavy metals like mercury into the interior of the cells (Solioz and Vulpe 1996).

### Calculation of maximum mercury internalization and mercury fixing constant

The data using internalized mercury/g dry‐weight versus incubation duration (h) for initial mercury concentration C_0_ = 1000 *μ*g L^−1^ (left *y*‐axis) and C_0_ = 50,000 *μ*g L^−1^ (right *y*‐axis) can be fitted by the functions *y *= (33.4618 *x*) / (6.5369 +  *x*) (*r* ^2^ = 0.97)} and *y *= (1977.0183 *x*) / (3.1679 +  *x*) (*r* ^2^ = 0.97), respectively, where *y* = accumulated mercury (mg/g dry‐weight) and *x* = duration of incubation (h) (Fig. 5B). All the functions are valid for denominators ≠ 0. Both functions can be generalized as functions of the type

(2) $$y\;=\;\frac{\left(B_{\max}\;\times \;x\right)}{\left(K_{d}\;+\;x\right)}$$

where *y* = accumulated/fixed Hg (mg/g dry‐weight biomass), *B*_max_ = maximum accumulation/fixing (mg/g dry‐weight biomass, see saturation levels), *K*_d_ =  fixing constant, *x* = duration of incubation (h), and *K*_d_ + *x* ≠ 0. Thus, for an application *C*_0_ = 1000 *μ*g L^−1^ we can find *B*_max_ = 33.468 mg/g dry‐weight reached within 36.2 h and *K*_d_ = 6.5369. At higher concentrations of applied mercury B_max_ can apparently increase by speeding up the transformation of ionic mercury into metallic mercury. Indeed, for *C*_0_ = 50,000 *μ*g L^−1^ we find *B*_max_ = 1,977.018 mg/g dry‐weight within 13.9 h and a lower fixing constant *K*_d_ = 3.1679. However, an intrinsic stability constant of mercury‐ligand fixing on a molar basis could not be calculated, as the molecular mass of the putative enzymatic fixing/binding molecule remains unknown (see below). The best fit by a ligand‐binding one‐site saturation function (*r* ^2^  = 0.99, Fig. 5B; eq. (2)) suggests that the internalization of mercury took place by binding at a single site of only one‐type of molecules, for example, at mercury reductase enzyme molecules, similar to those in *Penicillium* sp. MR‐2 strain (Tezuka and Someya 1990) and *Cephalosporium tabacum* strain F2 (Baojun et al. 1995). The mercury remained fixed to the EH8 biomass even after its transfer to mercury‐free aqueous medium at the same pH. Only a negligible dissociation of fixed mercury was observed by increasing acidity (only 2–3% release at pH 6, max. 9% release at pH 4) or alkalinity (only 2–3% release at pH 8, max. 9% release at pH 9) of water below or above optimum neutral pH. The optimum pH for mercury reductase enzymes in two other fungi was also found to be near neutral range, pH 7–8 (Tezuka and Someya 1990; Baojun et al. 1995).

### Localization of mercury by scanning electron microscopy (SEM) and energy‐dispersive x‐ray (EDX) analysis

**Figure 6:**
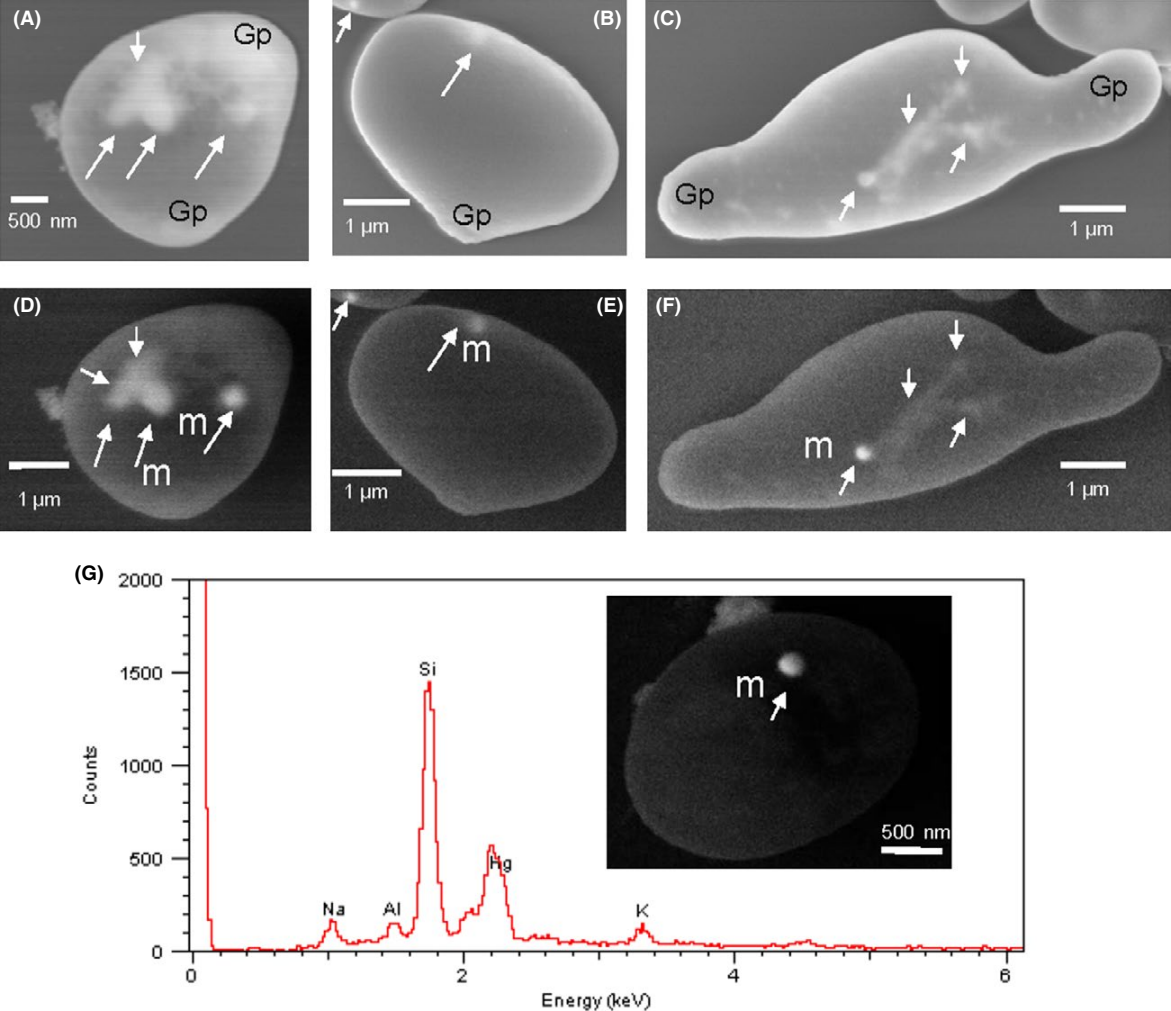
Intracellular accumulation, fixing, and reduction of ionic Hg in germinating EH8 sporangiospores. SEM images A–B and D–E show initial states of germination. C and F exhibit physiologically active elongating hypha from initiation of germination poles (Gp) after 48 h incubation with 50,000 *μ*g L^−1^ Hg(II). D–F are recorded by scanning the high energetic back‐scattered electrons from approx. 0.2–1 *μ*m depth of the same regions of A–C, respectively. In A, C–D and F, the elongated spots (see arrow) show the areas of mercury‐associated structures and release of spherical droplets (m) as typical formations of elemental mercury. (G) Electron‐dispersive x‐ray element analysis of a spherical metal droplet (m, size ca. 0.05 *μ*m^2^), for example, in one of the germinating EH8 sporangiospores, confirmed strong presence of mercury with HgM_*α*_ peak at 2.195 keV, whereby the spherical metallic droplet formations at approx. 0.6 *μ*m depth suggest intracellular deposition of elemental mercury (see Naidich et al. 1973).

SEM of mercury‐treated EH8 spores at various germination stages revealed intracellular mercury in clouds of associated components as well as deposition of spherical droplets of several hundred nanometer diameters (Fig. 6A–G). Additional scanning of higher energy back‐scattered electrons from approx. 0.2–1 *μ*m deeper intracellular layers by SEM detected the metallic‐shiny nature of these spherical droplets. Accumulation of mercury occurred even in active elongated hyphal areas (Fig. 6G) of germinating sporangiospores (see below). The larger metallic‐shiny spherical droplets (m) were also visible mainly at intracellular areas located most distal from the germination pole (Gp). Due to high water–metal interfacial tension of metallic mercury, it cannot wet the cellular surfaces (Naidich et al. 1973). Therefore, when elemental Hg is deposited in cytoplasm it can collect into spheres; if agitated, they can disperse into numerous tiny spherules, some of which may combine to form larger spherical droplets (Naidich et al. 1973). We observed the same phenomenon here. EDX analysis of such a metallic droplet (area size ca. 0.05 *μ*m^2^) at about 0.6 *μ*m depth inside a spore exhibited the characteristic major M shell signal peak (HgM_*α*_) of elemental Hg at 2.195 keV (Fig. 6G). The other low‐intensity L shell signal peaks (HgL_*α*_ at ~9.9 keV and HgL_*β*_ at ~11.8 keV) characteristic of mercury was not further measured because only ionic Hg was applied to incubation water containing the activated spores. The Si, Al, Na, and K peaks detected could be due to the sample preparations on glass slide or due to the incubation medium components. Thus, the intracellular formation of spherical shiny metallic droplets from applied ionic mercury and the proof that such a metallic droplet indeed contains mercury together confirm the unique property of EH8 in inactivating toxic ionic mercury by reduction into elemental mercury (Hg^0^). As no P‐peak was detected in the EDX spectrum of mercury‐containing droplets of EH8, the polyphosphate‐containing inclusions (Kiyono et al. 2003) cannot play any role for mercury accumulation and fixing/binding in EH8. In contrast to EH8, the intracellular accumulation of another non‐IIb group metal “bismuth” by the fungus *Fusarium* sp. strain BI occurred only in phosphorus‐rich inclusions (Dodge and Wackett 2005).

The formation of elemental Hg by reduction in Hg(II) was previously demonstrated in two terrestrial fungal strains *Penicillium* sp. MR‐2 strain (Tezuka and Someya 1990) and *Cephalosporium tabacum* strain F2 (Baojun et al. 1995) as well as in biofilms of *Pseudomonas putida* Spi3 (Wagner‐Döbler et al. 2000). However, using molecular biological methods, sequences of putative mercury‐reductase MerA gene were also detected in two terrestrial pathogenic fungi *Grossmania clavigera* strain kw1407/UMAH 11150 (DiGuistini et al. 2011) and *Cordyceps militaris* strain CM01 (Zheng et al. 2011). Two other fungi *Candida albicans* and *Saccharomyces cerevisiae* were also able to reduce low amounts of ionic Hg, but only in extracellular medium (Yannai et al. 1991). In contrast to EH8, in other fungi there is evidence of extracellular adsorption of Hg(II) to cell walls, namely in *Rhizopus arrhizus* (Özer et al. 1997) and *Phanerochaete chrysosporium* (Saglam et al. 2002). The removal of mercury solely by passive biosorption in EH8 could be excluded because of the fact that the uptake and distribution of mercury took place in the gradually growing germinating sporangiospores in high amounts (Fig. 6).

A positive correlation between mercury contents and catalase activity as well as a significant relationship between mercury levels and sulfhydryl contents in higher fungi, of which 83% were protein bound, were demonstrated (Kojo and Lodenius 1989). It was reported that sulfhydryl compounds (glutathione, cysteine) increased the protection and mercury tolerance in *Aspergillus niger*, a key feature that was enhanced after growth of this fungus on reduced sulfur, but not on sulfate, whereas the fungi *Rhizoctonia solani* and *Pythium ultimum* were not protected (Ashworth and Amin 1964). Similar to *Aspergillus niger*, high mercury‐tolerant EH8 in biofilms was also adapted to S‐reducing sulfidic spring water of Marching. Mercury resistance among some other strains of various fungi *Botrytis cinerea*,* Penicillium notatum*,* Sclerotinia fructicola*,* Stemphylium sarcinaeforme*, and *Saccharomyces cerevisiae* was observed, but yeast required methionine for mercury tolerance (Singh and Sherman 1974). However, intracellular non‐protein thiols were suggested not to be involved in mercury resistance of diverse fungi (Greenaway and Ward 1977), which supports our interpretations for EH8 (see below).

The time‐dependent mercury‐removal and internalization/fixing functions as detected by the kinetic study (Fig. 5) are in line with our view of intracellular coexistence of mercury‐binding and ‐reduction sites in EH8 (Fig. 6). The intermediate binding of ionic mercury in EH8 for chemical reduction can be mediated by a thiol ligand located, for example, in cysteine‐containing peptides or proteins (Pazirandeh et al. 1998; Saglam et al. 2002; Barkay et al. 2003), similar to mercury‐reductase enzymes found in some other fungi (Tezuka and Someya 1990; Baojun et al. 1995) or their associated proteins. Such an enzyme/protein ligand can contain at least two thiolates (Lian et al. 2014) and catalyze a two electron reduction of Hg(II) utilizing reduced NADPH (dihydronicotinamide adenine dinucleotide phosphate) or NADH (dihydronicotinamide adenine dinucleotide) according to the biochemical reaction: Hg(SR)_2_ + NADPH (or NADH)‐> Hg^0^ + NADP^+^ (or NAD^+^) + 2 RSH (Schiering et al. 1991; Baojun et al. 1995), via intermediary redox reactions of flavin adenine dinucleotide (FAD; Lian et al. 2014). Thus, this completion of biochemical reactions by enzymatic binding, reduction, and release of Hg^0^ as well as restoration of enzyme's active sites can involve two thiol function groups at a single binding site of Hg(II) (Raybuck et al. 1990). It should be borne in mind that EH8 was isolated from a cold sulfidic‐reducing environment, where it could adapt and develop high intracellular‐specific S‐reducing enzymes/proteins and biochemical mechanism for the reduction of oxidized Hg^2+^. It is plausible because another strain EH5 of the same fungus from a sulfidic‐reducing spring “Irnsing‐H_2_S” showed high expression of glutathione‐S‐transferase enzymes after growth on thiosulfate (Hoque et al. 2007). As the formation of elemental Hg can reduce the toxicity of Hg^2+^, this could be the main reason of high Hg tolerance and resistance in EH8, so that it can remove ionic mercury even in large amounts from bulk amount of water. Further cellular protection and resistance by EH8 could be achieved by uptake and secured placement of mercury deposits far away from sensitive germination points (Fig. 6D–F). Although by using ICP‐MS we detected mercury in Marching spring biofilms and water, the stereomicroscopic observations did not reveal any mercury droplets in biofilms obviously because of low concentrations of mercury in spring water and biofilm.

**Figure 7:**
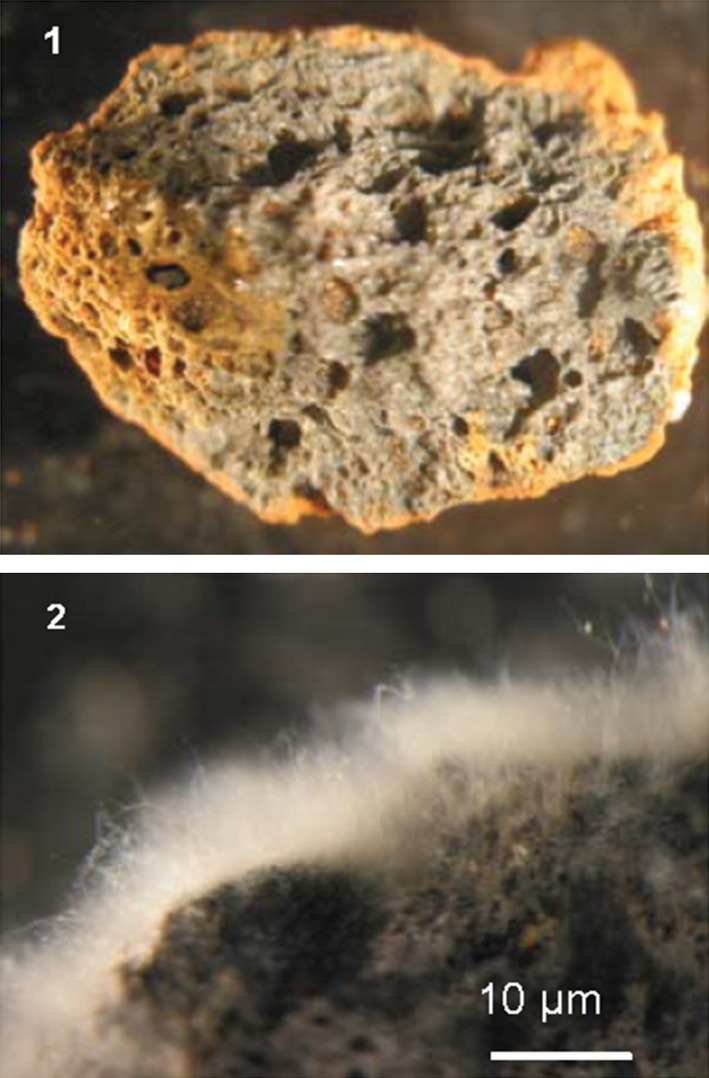
Growth of EH8 on expanded clay. (1) Sterilized expanded clay (4–8 mm diameter) before cultivation, (2) EH8's biofilm cultivated on expanded clay for in situ or ex situ treatment of mercury‐contaminated water even at low‐temperature sulfur‐reducing conditions.

Removal of mercury by microbially active metabolic intracellular accumulation rather than adsorption was advocated by Zhao et al. (2005) as the best method of mercury removal because the low bioavailability of toxic metals in the environment due to the tight metal complexation may be compensated by microbial metabolic processes for accumulation. Similarly, simultaneously expressed intracellular merT, merP, and metallothionein (mt) proteins during mercury metabolism enhanced mercury accumulation in genetically engineered *E. coli* JM109 (Zhao et al. 2005). EH8 fulfills the best condition of mercury removal by intracellular accumulation (see above) as advocated by Zhao et al. (2005), whereby the production of huge numbers of sporangiospores from EH8's sporangia, and their germination on low‐cost expanded clay in liquid media can be conveniently carried out (Fig. 7). Aquatic *M. hiemalis* strains from cold sulfidic springs are able to grow even at temperatures as low as 0.3°C (Hoque 2003). As compared to published bacterial and fungal mercury detoxification methods, our discovery can enable high mercury removal even at low‐temperature sulfur‐reducing conditions producing low amount of waste. In situ mercury removal can be conveniently carried out using filter bags filled with low biomass of EH8's activated sporangiospores or EH8's biofilms grown on expanded clay depending on environmental conditions or ex situ by passing contaminated water through columns packed with EH8 biofilms grown on expanded clay. Accumulated mercury can be easily physically separated by volatilization or washed out chemically from expanded clay, which can be recycled again after cleaning.

## Conclusions

Results revealed that historical mercury stress can be recorded by a microbial biofilm of a spring even after years, analogous to the function of a data logger. *M. hiemalis* EH8 from Marching Spring biofilm showed the unique ability to incorporate and detoxify ionic mercury by intracellular reduction into metallic mercury during the elongating active growth phase of germination. To the best of our knowledge, EH8 is the only natural aquatic eukaryotic microbe so far known to be capable of high mercury removal even at lower temperature sulfidic conditions by its intracellular accumulation and deposition, leaving only negligible waste. Biofilms of cold sulfidic‐reducing springs can be promising bioresources not only for purification of mercury‐contaminated water (Fritscher et al. 2006) but also for some other useful biotechnologies.

## Conflict of Interest

No conflict of interest.

## Supporting information


**Table S1**. Removal efficiency of Zn(II) and Cd (II) by biofilms and crucial fungal cultures from sulfidic springs. The mean percentages of Zn(II) and Cd (II) removal from water were calculated after measurements following applications of 1000 µg/L to biofilms (Bf) and corresponding fungal cultures (F). The matched data of significant values from biofilms and fungus of selected springs are shown in bold faces, whereas contrasting data are given in italics. The standard deviations of measurements (*n* = 3) were maximum 5%. Statistically significant higher values than control ones (low anthropogenic‐influenced control spring: Teugn) are marked by * sign (Student's one‐sided t‐test, *P* ≤ 0.05).
