# Supplementary material for: A new mercury‐accumulating Mucor hiemalis strain EH8 from cold sulfidic spring water biofilms
Source: Microbiologyopen. 2016 May 13;5(5):763–81. doi: 10.1002/mbo3.368 (PMC5061714; doi:10.1002/mbo3.368)
Supplement: Supplementary file 1 — Table S1. Removal efficiency of Zn(II) and Cd (II) by biofilms and crucial fungal cultures from sulfidic springs. The mean percentages of Zn(II) and Cd (II) removal from water were calculated after measurements following applications of 1000 µg/L to biofilms (Bf) and corresponding fungal cultures (F). The matched data of significant values from biofilms and fungus of selected springs are shown in bold faces, whereas contrasting data are given in italics. The standard deviations of measurements (n = 3) were maximum 5%. Statistically significant higher values than control ones (low anthropogenic‐influenced control spring: Teugn) are marked by * sign (Student's one‐sided t‐test, P ≤ 0.05). [file MBO3-5-763-s001.doc]

**Supplement**

TABLE S1: Removal efficiency of Zn(II) and Cd (II) by biofilms and crucial fungal cultures from sulfidic springs. The mean percentages of Zn(II) and Cd (II) removal from water were calculated after measurements following applications of 1,000 µg/l to biofilms (Bf) and corresponding fungal cultures (F). The matched data of significant values from biofilms and fungus of selected springs are shown in bold faces, whereas contrasting data are given in italics. The standard deviations of measurements (n = 3) were maximum 5%. Statistical significant higher values than control ones (low anthropogenic-influenced control spring: Teugn) are marked by * sign (Student´s one-sided t-test, p ≤ 0.05).

| Spring/Fungus | Zn  Bf F | Cd  Bf F |
| --- | --- | --- |
| Irnsing H_2_S: *Mucor hiemalis* EH5 | 54.6 52.5 | 41.5 10.3 |
| Marching: *Mucor hiemalis* EH8 | 48.1 46.2 | *95.5 6.0* |
| Bad Abbach: *Geotrichum candidum* | *37.9 0* | *36.6 0* |
| Sippenauer Moor: *Mucor hiemalis* EH9 | 51.1 49.2 | 47.9 17.6 |
| Schwandorf: *Mucor hiemalis* EH12 | 32.8 30.7 | 49.5 36.2 |
| Wildbadkreuth: *Polyphagus euglenea* | *28.9 0* | *41.5 0* |
| Quarzitwerk: *Mucor hiemalis* EH10 | **73.9* 71.3*** | **93.8 91.3*** |
| Pilzweg: *Fusarium avenaceum* | 35.6 33.0 | **59.8** **56.0*** |
| Künzing: *Mucor hiemalis* EH11 | **86.4* 83.6*** | **96.6 80.3*** |
| Bad Höhenstadt: *Mucor hiemalis* EH4 | 24.8 24.8 | 53.0 53.0 |
| Bad Gööging: *Mucor hiemalis* EH6 | 31.1 27.4 | *88.7 44.0* |
| Teugn:  *Mucor hiemalis* EH7 | 51.7 50.0 | *96.9 22.0* |
